# Supplementary material for: Iterative improvement in the automatic modular design of robot swarms
Source: PeerJ Comput Sci. 2020 Dec 7;6:e322. doi: 10.7717/peerj-cs.322 (PMC7924708; doi:10.7717/peerj-cs.322)
Supplement: Supplemental Information 3 [file peerj-cs-06-322-s003.zip › argos3/doc/api/standalone/a00455_source.html]

ARGoS: plugins/robots/foot-bot/simulator/dynamics2d\_footbot\_model.h Source File


- Main Page
- Related Pages
- Namespaces
- Classes
- Files

- File List
- File Members

# plugins/robots/foot-bot/simulator/dynamics2d\_footbot\_model.h

Go to the documentation of this file.

```
00001 
00007 #ifndef DYNAMICS2D_FOOTBOT_MODEL_H
00008 #define DYNAMICS2D_FOOTBOT_MODEL_H
00009 
00010 namespace argos {
00011    class CDynamics2DDifferentialSteeringControl;
00012    class CDynamics2DGripper;
00013    class CDynamics2DGrippable;
00014 }
00015 
00016 #include <argos3/plugins/simulator/physics_engines/dynamics2d/dynamics2d_multi_body_object_model.h>
00017 #include <argos3/plugins/simulator/physics_engines/dynamics2d/dynamics2d_differentialsteering_control.h>
00018 #include <argos3/plugins/robots/foot-bot/simulator/footbot_entity.h>
00019 
00020 namespace argos {
00021 
00022    class CDynamics2DFootBotModel : public CDynamics2DMultiBodyObjectModel {
00023 
00024    public:
00025 
00026       CDynamics2DFootBotModel(CDynamics2DEngine& c_engine,
00027                               CFootBotEntity& c_entity);
00028       virtual ~CDynamics2DFootBotModel();
00029       
00030       virtual void MoveTo(const CVector3& c_position,
00031                           const CQuaternion& c_orientation);
00032 
00033       virtual void Reset();
00034 
00035       virtual void CalculateBoundingBox();
00036 
00037       virtual void UpdateFromEntityStatus();
00038 
00039       void UpdateOriginAnchor(SAnchor& s_anchor);
00040 
00041       void UpdateTurretAnchor(SAnchor& s_anchor);
00042 
00043       void UpdatePerspectiveCameraAnchor(SAnchor& s_anchor);
00044 
00045       inline cpBody* GetActualBaseBody() {
00046          return m_ptActualBaseBody;
00047       }
00048 
00049       inline const cpBody* GetActualBaseBody() const {
00050          return m_ptActualBaseBody;
00051       }
00052 
00053       inline cpBody* GetActualGripperBody() {
00054          return m_ptActualGripperBody;
00055       }
00056 
00057       inline const cpBody* GetActualGripperBody() const {
00058          return m_ptActualGripperBody;
00059       }
00060 
00061    private:
00062 
00063       void TurretPassiveToActive();
00064       void TurretActiveToPassive();
00065 
00066    private:
00067 
00068       CFootBotEntity&         m_cFootBotEntity;
00069       CWheeledEntity&         m_cWheeledEntity;
00070       CGripperEquippedEntity& m_cGripperEntity;
00071 
00072       CDynamics2DDifferentialSteeringControl m_cDiffSteering;
00073       CDynamics2DGripper*                    m_pcGripper;
00074       CDynamics2DGrippable*                  m_pcGrippable;
00075 
00076       cpFloat  m_fMass;
00077       cpShape* m_ptBaseShape;
00078       cpBody*  m_ptActualBaseBody;
00079       cpBody*  m_ptActualGripperBody;
00080       cpBody*  m_ptControlGripperBody;
00081       cpConstraint* m_ptGripperControlAngularMotion;
00082       cpConstraint* m_ptBaseGripperLinearMotion;
00083       cpConstraint* m_ptBaseGripperAngularMotion;
00084 
00085       const Real* m_fCurrentWheelVelocity;
00086 
00087       UInt8 m_unLastTurretMode;
00088       Real m_fPreviousTurretAngleError;
00089 
00090    };
00091 
00092 }
00093 
00094 #endif
```

---

Generated on 10 Jul 2018 for ARGoS by 
 1.6.1
